# Linguistic Validation of the Canine Brief Pain Inventory (CBPI) for Global Use

**DOI:** 10.3389/fvets.2021.769112

**Published:** 2021-11-29

**Authors:** Jane R. Wells, Alyson L. Young, Alexandra Crane, Hilde Moyaert, Gina Michels, Andrea Wright

**Affiliations:** ^1^Patient-Centered Outcomes, Adelphi Values Ltd, Adelphi Mill, Cheshire, United Kingdom; ^2^TransPerfect, New York, NY, United States; ^3^Veterinary Medicine Research and Development, Zoetis Belgium SA, Zaventem, Belgium; ^4^Veterinary Medicine Research and Development, Zoetis Inc, Kalamazoo, MI, United States; ^5^Outcomes Research, Zoetis Inc, Parsippany, NJ, United States

**Keywords:** linguistic validation, canine, translation, Canine Brief Pain Inventory, pain

## Abstract

A valid and reliable quantitative measure of chronic pain is essential for developing and evaluating interventions that aim to treat pain. In dogs, the Canine Brief Pain Inventory (CBPI) was originally adapted from a human measure, the Brief Pain Inventory, to assess owner-perceived pain and the impact of such pain on a dog's daily functioning. To be reliable and valid, data collected using a translated instrument should have evidence it is an accurate representation of the original instrument and is culturally appropriate for use in the intended context. To achieve this, instruments should undergo a rigorous translation process and be debriefed in the intended population of use. The CBPI is widely accepted and has been fully validated for use in US-English, Swedish, Italian, and French (France); further translation and validation of the CBPI is required to increase access to and use in other languages and countries. The objective of this study was to linguistically validate the CBPI for global use (Australia, China, Germany, Hungary, Ireland, Japan, Netherlands and Portugal). In cognitive debriefing with a representative sample of dog owners in the target countries it was confirmed that the translations of the CBPI adequately convey the concepts in the original US-English version and that items are easily understood by dog owners. The results of the linguistic validation process thus produced measures that are conceptually equivalent to the original US-English-language CBPI and are culturally appropriate for use in the target countries.

## Introduction

The often complex, multidimensional, and subjective nature of pain makes it difficult to assess. Valid and reliable measures of pain are therefore essential for developing and evaluating interventions (e.g., drugs or surgical procedures) that aim to treat such pain (1, 2). In dogs, pain assessment tools have been developed and validated to capture owner-perceived pain in canines, specifically for osteoarthritis (2, 3). One such assessment is the Canine Brief Pain Inventory (CBPI), which has been adapted from the human measure, the Brief Pain Inventory (BPI) (4–6), to assess a dog's pain severity and pain interference in their daily activities. The CBPI draws on the owner's understanding and awareness of their dog's behavioral changes that might indicate pain (2, 7). Items for the CBPI were generated in interviews and cognitively debriefed with dog owners, and psychometric validation has demonstrated adequate construct validity and criterion validity for measuring a dog's pain (2, 7), as well as the ability to detect clinically important changes in a dog over time (8).

For broad adoption, translation of the CBPI is an essential step to ensure its use as a standardized, valid, and reliable measure in clinical research and practice. Linguistic validation is the process by which translated measures are assessed for cultural appropriateness and conceptual equivalence, and to ensure that content validity of the original measure has not been affected by translation (9). This mitigates the risk of data being invalid due to ambiguous or incorrect translation of items or instructions, and better ensures that any differences in responses between contributing populations are due to differences between groups rather than differences in how the data were collected (9, 10). Evidence of linguistic validation means that a translated instrument can more confidently be used in populations where the participant's first language differs from that of the original instrument and for the resulting data to be aggregated and compared across cultures and languages because the same measure has been used (9, 10).

Since its initial development in US English, the CBPI has been translated and validated for use in Swedish (11), French (12) and Italian (13), with initial psychometric validation of the French CBPI indicating evidence of construct validity, convergent validity, and strong internal consistency (12). The aim of this study was to develop conceptually equivalent and culturally relevant versions of the CBPI for use in Australia (English), Ireland (English), China (Chinese, simplified), Japan (Japanese), Hungary (Hungarian), Germany (German), Portugal (Portuguese), and the Netherlands (Dutch), as a first step in establishing the CBPI as an internationally validated owner-reported measure for evaluating pain in dogs.

## Materials and Methods

Using established methods from the World Health Organization (WHO) and the International Society of Pharmoeconomics and Outcomes Research (ISPOR) (9, 10, 14, 15), the CBPI was translated into Australian-English (Australia), Simplified Chinese (China), Dutch (Netherlands), German (Germany), Hungarian (Hungary), Irish-English (Ireland), Japanese (Japan), and Portuguese (Portugal) and cognitively debriefed with a sample of dog owners from each country to evaluate their understanding of the measure and confirm that the translations of the CBPI adequately convey the concepts in the original US-English version. Permission to translate the CBPI was given by the developer.

**Figure 1:**
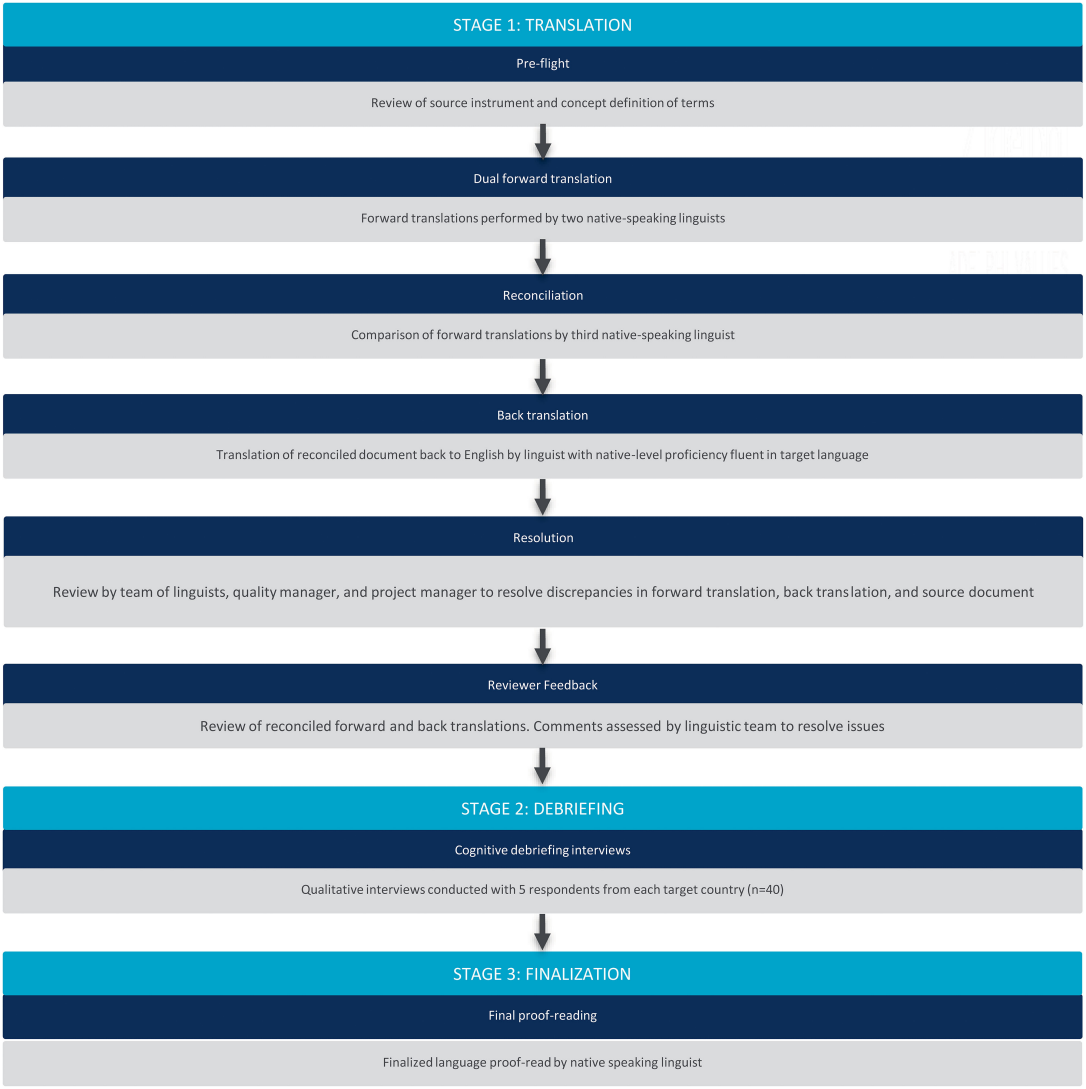
Translation and linguistic validation process.

Prior to translation, the CBPI was divided into key terms and phrases, with definitions for each concept, to ensure the intended meaning was captured in the translation. All translators (linguists) aimed for conceptual equivalence to the source and cultural appropriateness for the target country. The translation and validation process is outlined in Figure 1.

Just as in the original CBPI, all translated versions consist of four items pertaining to the severity of pain evident in a dog (pain severity domain) and six items relating to pain interference with daily activities (pain interference domain). Items in both domains are measured using a 0–10 numerical rating scale. For the pain severity domain items, 0 = no pain and 10 = extreme pain. For the interference domain items, 0 = does not interfere and 10 = completely interferes. Means values for each set of responses are calculated to determine the pain severity and pain interference scores, respectively. A single global quality of life (QoL) item is included to obtain the owner's overall assessment of the dog's QoL status. This item assesses QoL using a 5-point categoric response scale (1 = poor; 2 = fair; 3 = good; 4 = very good; and 5 = excellent) (2, 8).

### Translation Process

Two native-speaking linguists of the target language independently performed forward translations on the CBPI. A third independent native-speaking linguist compared the two forward translations to identify any discrepancies or cultural differences in order to create a unified forward translation (reconciliation). An independent linguist with native-level English proficiency and fluent in the target language then translated the reconciled document back to the source language (US-English) using the forward translation as source material (back translation). Following this, an independent review was performed by a team of linguists (both English and target languages), a quality manager, and a project manager to examine and resolve any discrepancies between the forward translation, back translation, and source document (resolution). The reconciled forward and back translations were then reviewed to ensure clarity of wording and intended measurement of concepts were captured in the translation. Reviewer comments were then assessed by a linguist to resolve any issues with the translation.

For the Australian-English and Irish-English versions, the source content was first localized to local language conventions to produce a forward “translation” and then reviewed to ensure clarity of wording and intended measurement concepts were captured (10).

### Cognitive Debriefing Interviews

Following translation, cognitive interviews were conducted with dog owners to determine if the CBPI instructions, response options, and items were clear, unambiguous, and relevant to participants. Participants included five dog owners from each target country (*n* = 40) (9). Participants were asked to read through the CBPI, discuss how they understood each item and the associated response options, and suggest potential alternative wording. Findings were used to make further adjustments to the translations.

For the German, Chinese, and Japanese translations, the back translation was reviewed a second time following updates from the cognitive interviews. This was done to ensure that any additional modifications made at this stage were conceptually equivalent to the source material. Conceptual equivalence was either confirmed by a linguist or further modifications were made to better align the translation with the source material.

### Finalization

In the last step, a native-speaking linguist of the target language proof-read the document, leading to the final translation for the target country.

## Results

The linguistic validation process led to Australian-English (Australia), Simplified Chinese (China), Dutch (Netherlands), German (Germany), Hungarian (Hungary), Irish-English (Ireland), Japanese (Japan), and Portuguese (Portugal) versions CBPI that are linguistically validated and conceptually equivalent to the original US English version. Example changes made throughout the linguistic validation process for the Portuguese translation can be found in Table 1.

**Table 1:** Examples of discrepancies and changes in the linguistic validation process for Portuguese.

**Source (US-En)**	**Forward translation and reconciliation**	**Back translation**	**Independent review (Resolution)**	**Reviewer feedback**	**Linguist feedback**	**Cognitive debrief**	**Linguist feedback**	**Updated back translation**
Rate your dog's pain	Consensus reached on forward translation: *Classifique a dor do seu cão*	Please rate your dog's pain	It is confirmed that the FT and BT are accurate reflections of the English source	Does this need to have ‘please’ for local reasons?	No, please is not needed and it is not included in the translation. Translation is correct as it is. BT updated to better reflect FT source: Rate your dog's pain	No difficulties reported	No revision needed	Rate your dog's pain
Fill in the oval next to the one number that best describes the pain at its worst in the last 7 days	Forward translations differed. Reconciled by third linguist: *Preencha o espaço oval a sequir ao número que melhor descreve a pior dor nos últimos 7 dias*	Fill in the oval space after the number that best describes the worst pain in the last 7 days	FT updated to match reference as requested: Preencha o oval junto ao número que melhor descreve o nivel de dor mais forte nos últimos 7 dias BT updated accordingly: Fill in the oval next to the one number that best describes the pain at its worst in the last 7 days	Agree	No revision needed	No difficulties reported	No revision needed. R4 suggested using the word “circulo” instead of “oval” but had no problem understanding	Fill in the oval next to the one number that best describes the pain at its worst in the last 7 days
No Pain	Forward translations differed. Reconciled by third linguist: *Nenhuma dor*	No pain	FT updated to match reference as requested: *Sem dor* BT updated accordingly: No pain	Agree	No revisions needed	No difficulties reported	No revisions needed	No pain
Does not interfere	Forward translations differed. Reconciled by third linguist: *Não interfere*	Does not interfere	FT updated to match reference as requested: *Não interferiu* No revision needed to BT	Agree	No revisions needed	No difficulties reported	No revisions needed	Does not interfere
Enjoyment of life	Forward translations differed. Reconciled by third linguist: *Apreciar a vida*	Enjoying life	FT updated to match reference as requested: *Aproveitamento da vida* BT updated accordingly: Enjoyment of life	Agree	No revisions needed	No difficulties reported	No revision needed. R4 had some difficulty understanding what enjoyment of life was, he wasn't sure if it was referring to general activities. He notes he had no trouble understanding the question. No changes needed	Enjoyment of life
Fair [“fair” = unsatisfactory]	Consensus reached on forward translation: *Razoável*	Reasonable	FT and BT translation updated to better reflect source as per concept. Deviation from reference file justified: *Insatisfatório* *Unsatisfactory*	Please confirm that this term works in the context of the response option	Yes, both are correct. Insatisfatório was used based on feedback and the concept definition. No revision needed	No difficulties reported	No revisions needed	Unsatisfactory

### Translation of the CBPI

In the dual forward translation, we found that the two independent linguists had similar views on the target language translations with the exception of the Portuguese, Chinese, and Japanese translations. Indeed, for the Japanese forward translations there were 18 items or instructions (out of a total 25) that differed between the two native-speaking linguists, while for the Chinese and Portuguese forward translations there were 16 and 13 items or instructions that differed, respectively. In the reconciliation process, the third independent native-speaking linguist reconciled these differences by either selecting the most appropriate translation (in consideration of the source statements) or proposing an alternative translation that better reflected the source materials.

In the resolution phase, the team of linguists, quality manager, and project manager identified 53 items or instructions that differed from the forward translation, back translation, and the source materials (Japanese: *n* = 16; Portuguese: *n* = 16; Chinese: *n* = 8; German: *n* = 7; Hungarian: *n* = 5; and Dutch: *n* = 1). For example, in the interference domain, the original item “enjoyment of life” was initially back translated from Portuguese to “enjoying life,” which was judged to have a different meaning and thus required re-translation. Another example was the use of the German word for “number” (“zahl”) rather than “response” (“antwort”) in the instructions for the overall QoL scale. This was revised to better reflect the original English version and intent.

In comparison of the back translation and the original US-English version, the reviewers identified the items or instructions that differed from the source material [Japanese: *n* = 7 instances; German: *n* = 4 instances; Dutch: *n* = 4 instances; Chinese (simplified): *n* = 2 instances; Portuguese: *n* = 2 instances; and Hungarian: *n* = 3 instances]. For three of items in the German translation and four items in the Dutch translation, the reviewer confirmed that while there was a slight deviation from English, the terms used reflected intention and therefore no revisions were needed. In the Hungarian back translation, the reviewer flagged use of the word “limited” in the anchors of the interference domain as inconsistent with the intended meaning of “interfere” in the original English version of the measure, which impacted three of the items/instructions. The anchors were re-translated to address this difference. For two items in the Portuguese translation, the reviewer asked for clarification on whether a specific term was required for local conventions; for example, whether “please” was needed for the item “[please] rate your dog's pain.” The linguist confirmed that this was not needed and back translation was updated to better reflect the source and forward translation. Similarly, in the Japanese translation, the reviewer asked for confirmation that a deviation in sentence structure for seven of the items was due to local language conventions or whether this could be updated to better reflect the source material. The linguist confirmed that Japanese grammar conventions differ from English and that sentence structure was correct for the target language and accurately reflected the source material.

In the Irish-English forward translation, the reviewer identified 12 revisions that appeared non-essential, of which one was deemed necessary by the linguist (i.e., changing the spelling of “kerb” to “curb”) and the forward translation was updated. The remaining 11 revisions focused on the use of Irish-English capitalization rules, which did not affect item understanding. Therefore, the original capitalization style was retained. No issues in translation were identified in the Australian-English version.

### Cognitive Debriefing of the CBPI Translations

Cognitive interviews were conducted with five dog owners from each of the target countries (*n* = 40) to evaluate their understanding and cultural appropriateness of the CPBI (9). Table 2 shows participant characteristics. In general, items were easily understood by the participants demonstrating the instructions, response options, and items were clear, unambiguous, and relevant to participants. In a few cases, participants suggested alternative words or phrases they felt either better reflected the sentiment of the item or sounded more natural in the context of the question. For example, in the Chinese translation, three participants reported difficulty understanding the term used for “average” in the instructions for rating average pain as this term is typically associated with calculations in Chinese language conventions. Suggestions were made to change the instruction wording to specify “in general” or “in most cases” and the instruction was subsequently updated to include the term “in general.” Additionally, in the Hungarian translation, two participants reported a dislike for the phrase “best describes” (“legjobban leírja”) in the instruction to “Fill in the oval next to the one number that best describes the pain…,” with one participant suggesting the alternative “best characterizes” (“legjobban jellemzi”) as a more natural phrase. With the exception of the Chinese translation, suggestions were deemed stylistic by the linguist and therefore the original translation was retained. No difficulties were reported in the Australian-English or Irish-English translations.

**Table 2:** Participant characteristics; cognitive interviews.

	**Australian (*n* = 5)**	**Chinese (*n* = 5)**	**Dutch (*n* = 5)**	**German (*n* = 5)**	**Hungarian (*n* = 5)**	**Irish (*n* = 5)**	**Japanese (*n* = 5)**	**Portuguese (*n* = 5)**	**Totals (*n* = 40)**
Female	3	3	3	3	2	2	3	3	22
Male	2	2	2	2	3	3	2	2	18
Age in years, mean (range)	45 (18–70)	45 (27–62)	42 (20–68)	53 (35–68)	56 (39–71)	47 (22–69)	52 (41–70)	45 (27–66)	48 (18–71)
Education level in years, mean (range)	12.2 (4–16)	12 (9–16)	12.6 (10–16)	10.2 (9–12)	13.8 (10–17)	13.2 (12–15)	14.4 (12–16)	13.8 (11–17)	12.8 (4–17)

A second review was performed by the reviewers following cognitive debriefing of the German, Chinese, and Japanese translations to ensure that any modifications made at this stage were conceptually equivalent with the source material. The reviewers identified three items in the German translation, one item in the Chinese translation, and six items in the Japanese translation where the meaning differed from the source material. For example, in the German translation, the item “rate your dog's pain” was back translated from “please evaluate your dog's pain,” which was deemed by the reviewer to deviate from the English source in such a way as to threaten the consistency between the languages and thus required re-translation. Additionally, the reviewer also questioned revisions made to the items “ability to run” and “ability to walk,” arguing that “gehen” implies a slower pace and therefore more adequately describes walking whereas “laufen” is more appropriate for describing running. In the Chinese translation, the reviewer requested that a more comparable term for “average” be used in place of “in general” for the instructions for the item assessing pain in the last 7 days as the latter does not imply the same meaning. This was subsequently re-translated to “average degree” to better match the source measure. While in the Japanese translation, the reviewer identified six items where word choices were inconsistent with the intended meaning in English and queried whether these were appropriate for the Japanese context (e.g., use of “applies” rather than “describes”). The appropriateness of the terms was confirmed by a linguist, and demonstrated in cognitive debriefing, and retained in the final version.

The final proofreading step led to minor changes in wording/grammar to provide clarity and comprehension.

## Discussion

As owner-reported outcome assessments become increasingly used in global clinical veterinary practice and research (16), the rigorous translation and linguistic validation of these measures is essential to ensure that data collected is valid and reliable. Linguistic validation is the process by which translated measures are assessed for content validity, and conceptual equivalence of language translation and cultural appropriateness for use in the target country or region (14). This process ensures that data collected using a translated instrument is an accurate representation of the original instrument and is culturally appropriate for use in the intended context. Rigorous translation and validation of an instrument also means that the results can more easily be directly compared cross-culturally (9, 10).

In this study, we describe the translation and linguistic validation of the CBPI to create versions in Australian and Irish-English, Simplified Chinese (China), Japanese (Japan), German (Germany), Dutch (Netherlands), Portuguese (Portugal), and Hungarian (Hungary). The CBPI has previously been translated and validated for use in Swedish (11), French (12), and Italian (13). Using established guidelines (9, 10, 14, 15), the CBPI was translated to the target languages using a rigorous process of forward and back translation and then cognitively debriefed with a representative sample of dog owners whose first language was one of the target languages. Cognitive debriefing feedback for each language of interest confirmed that the translations of the CBPI adequately convey the concepts in the original version of the questionnaire and that items are relevant and easily understood by dog owners. Only minor changes in terminology were made following cognitive debriefing to improve clarity, comprehension, and to account for cultural differences (e.g., use of the term “average” in the Chinese translation). This has resulted in translated measures that are conceptually equivalent to the original CBPI and culturally appropriate for use in the target countries.

The translation and linguistic validation of the CBPI into eight additional versions is an essential step in adapting the measure for use globally (Translated versions of the instruments are available as Supplementary Materials. Copyright is held by Dr. Dorothy Cimino Brown. Terms of use can be found at www.CanineBPI.com). Validation of an instrument, however, is an on-going process. It is also good practice that the translated instruments demonstrate that they measure what they are intended to measure (i.e., pain severity and pain interference) within the target populations. This can be achieved by demonstrating quantitative evidence of validity (e.g., construct validity, convergent validity) and reliability (e.g., internal consistency, test-retest reliability) of the measures. Further, for the translated instruments to be used to evaluate the effectiveness of interventions of chronic pain in dogs, they should also be able to detect clinically importance change, known as responsiveness. Quantitative assessments of cross-cultural validity of the translated measures can also be assessed to determine the extent to which instrument scores behave as expected in different populations, such as ethnic or language groups (17, 18). Initial psychometric validation has already been conducted for the French (12), Swedish (11), and Italian (13) versions of the CBPI, with the French version showing evidence of construct validity, convergent validity, and strong internal consistency of the measure. Next steps in this research could be for researchers to psychometrically test the newly translated versions to further demonstrate the validity of these instruments.

## Data Availability Statement

The raw data supporting the conclusions of this article will be made available by the authors, without undue reservation.

## Ethics Statement

Ethical review and approval was not required for the study on human participants in accordance with the local legislation and institutional requirements. Written informed consent for participation was not required for this study in accordance with the national legislation and the institutional requirements.

## Author Contributions

AC oversaw the linguistic validation process. JW, AY, GM, HM, and AW contributed to study conception and interpretation of results. JW, AY, AC, HM, GM, and AW contributed to writing this manuscript. All authors contributed to the article and approved the submitted version.

## Funding

This research was funded by Zoetis Inc.

## Conflict of Interest

JW and AY were employed by company Adelphi Values Ltd. AC was employed by company TransPerfect. GM, HM, and AW were employed by company Zoetis Inc.

## Publisher's Note

All claims expressed in this article are solely those of the authors and do not necessarily represent those of their affiliated organizations, or those of the publisher, the editors and the reviewers. Any product that may be evaluated in this article, or claim that may be made by its manufacturer, is not guaranteed or endorsed by the publisher.
