# Supplementary material for: Linguistic Validation of the Canine Brief Pain Inventory (CBPI) for Global Use
Source: Front Vet Sci. 2021 Nov 29;8:769112. doi: 10.3389/fvets.2021.769112 (PMC8666957; doi:10.3389/fvets.2021.769112)
Supplement: Supplementary file 1 [file Data_Sheet_1.PDF]

Canine Brief Pain Inventory (CBPI) - Australia

Description of Pain:
Rate your dog's pain.

1. Fill in the oval next to the one number that best describes the pain at its **worst** in the last 7 days.

   ○ 0 ○ 1 ○ 2 ○ 3 ○ 4 ○ 5 ○ 6 ○ 7 ○ 8 ○ 9 ○ 10

   No pain … Extreme pain

2. Fill in the oval next to the one number that best describes the pain at its **least** in the last 7 days.

   ○ 0 ○ 1 ○ 2 ○ 3 ○ 4 ○ 5 ○ 6 ○ 7 ○ 8 ○ 9 ○ 10

   No pain … Extreme pain

3. Fill in the oval next to the one number that best describes the pain at its **average** in the last 7 days.

   ○ 0 ○ 1 ○ 2 ○ 3 ○ 4 ○ 5 ○ 6 ○ 7 ○ 8 ○ 9 ○ 10

   No pain … Extreme pain

4. Fill in the oval next to the one number that best describes the pain as it is **right now**.

   ○ 0 ○ 1 ○ 2 ○ 3 ○ 4 ○ 5 ○ 6 ○ 7 ○ 8 ○ 9 ○ 10

   No pain … Extreme pain

Description of Function:
Fill in the oval next to the one number that best describes how during the past 7 days **pain has interfered** with your dog's:

5. **General activity**

   ○ 0 ○ 1 ○ 2 ○ 3 ○ 4 ○ 5 ○ 6 ○ 7 ○ 8 ○ 9 ○ 10

   Does not interfere … Completely interferes

6. **Enjoyment of life**

   ○ 0 ○ 1 ○ 2 ○ 3 ○ 4 ○ 5 ○ 6 ○ 7 ○ 8 ○ 9 ○ 10

   Does not interfere … Completely interferes

7. **Ability to rise to standing from lying down**

   ○ 0 ○ 1 ○ 2 ○ 3 ○ 4 ○ 5 ○ 6 ○ 7 ○ 8 ○ 9 ○ 10

   Does not interfere … Completely interferes

8. **Ability to walk**

   ○ 0 ○ 1 ○ 2 ○ 3 ○ 4 ○ 5 ○ 6 ○ 7 ○ 8 ○ 9 ○ 10

   Does not interfere … Completely interferes

9. **Ability to run**

   ○ 0 ○ 1 ○ 2 ○ 3 ○ 4 ○ 5 ○ 6 ○ 7 ○ 8 ○ 9 ○ 10

   Does not interfere … Completely interferes

10. **Ability to climb up (for example stairs or curbs)**

    ○ 0 ○ 1 ○ 2 ○ 3 ○ 4 ○ 5 ○ 6 ○ 7 ○ 8 ○ 9 ○ 10

    Does not interfere … Completely interferes

Overall Impression:

11. Fill in the oval next to the one response that best describes your dog's overall quality of life over the last 7 days.

    ○ Poor ○ Fair ○ Good ○ Very Good ○ Excellent

Canine Brief Pain Inventory (CBPI) - China

疼痛描述：

对您爱犬的疼痛进行评级。

1. 将最能描述过去 7 天最严重疼痛的数字旁边的椭圆形涂色。○0 ○1 ○2 ○3 ○4 ○5 ○6 ○7 ○8 ○9 ○10

没有疼痛 极度疼痛

2. 将最能描述过去 7 天最轻度疼痛的数字旁边的椭圆形涂色。○0 ○1 ○2 ○3 ○4 ○5 ○6 ○7 ○8 ○9 ○10

没有疼痛 极度疼痛

3. 将最能描述过去 7 天疼痛平均程度的数字旁边的椭圆形涂色。○0 ○1 ○2 ○3 ○4 ○5 ○6 ○7 ○8 ○9 ○10

没有疼痛 极度疼痛

4. 将最能描述当前疼痛的数字旁边的椭圆形涂色。○0 ○1 ○2 ○3 ○4 ○5 ○6 ○7 ○8 ○9 ○10

没有疼痛 极度疼痛

功能描述：

将最能描述过去 7 天的疼痛如何干扰您爱犬如下情况的数字旁边的椭圆形涂色：

5. **一般活动** ○0 ○1 ○2 ○3 ○4 ○5 ○6 ○7 ○8 ○9 ○10

没有干扰 完全干扰

6. **享受生活** ○0 ○1 ○2 ○3 ○4 ○5 ○6 ○7 ○8 ○9 ○10

没有干扰 完全干扰

7. **从躺下到起身站立的能力** ○0 ○1 ○2 ○3 ○4 ○5 ○6 ○7 ○8 ○9 ○10

没有干扰 完全干扰

8. **行走能力** ○0 ○1 ○2 ○3 ○4 ○5 ○6 ○7 ○8 ○9 ○10

没有干扰 完全干扰

9. **奔跑能力** ○0 ○1 ○2 ○3 ○4 ○5 ○6 ○7 ○8 ○9 ○10

没有干扰 完全干扰

10. **攀爬能力（例如楼梯或路缘）** ○0 ○1 ○2 ○3 ○4 ○5 ○6 ○7 ○8 ○9 ○10

没有干扰 完全干扰

总体印象：

11. 将最能描述过去 7 天您爱犬的总体生活质量的选项旁边的椭圆形涂色。 ○差 ○一般 ○好 ○很好 ○优

Canine Brief Pain Inventory (CBPI) - Germany

Beschreibung des Schmerzes:

Bewerten Sie den Schmerz Ihres Hundes.

1. Füllen Sie das Oval neben derjenigen Zahl aus, welche den **schlimmsten** Schmerz in den letzten 7 Tagen am besten beschreibt.

   ⬭ 0 ⬭ 1 ⬭ 2 ⬭ 3 ⬭ 4 ⬭ 5 ⬭ 6 ⬭ 7 ⬭ 8 ⬭ 9 ⬭ 10

   Kein Schmerz — Extremer Schmerz

2. Füllen Sie das Oval neben derjenigen Zahl aus, welche den **geringsten** Schmerz in den letzten 7 Tagen am besten beschreibt.

   ⬭ 0 ⬭ 1 ⬭ 2 ⬭ 3 ⬭ 4 ⬭ 5 ⬭ 6 ⬭ 7 ⬭ 8 ⬭ 9 ⬭ 10

   Kein Schmerz — Extremer Schmerz

3. Füllen Sie das Oval neben derjenigen Zahl aus, welche den **durchschnittlichen** Schmerz in den letzten 7 Tagen am besten beschreibt.

   ⬭ 0 ⬭ 1 ⬭ 2 ⬭ 3 ⬭ 4 ⬭ 5 ⬭ 6 ⬭ 7 ⬭ 8 ⬭ 9 ⬭ 10

   Kein Schmerz — Extremer Schmerz

4. Füllen Sie das Oval neben derjenigen Zahl aus, welche den Schmerz **jetzt gerade** am besten beschreibt.

   ⬭ 0 ⬭ 1 ⬭ 2 ⬭ 3 ⬭ 4 ⬭ 5 ⬭ 6 ⬭ 7 ⬭ 8 ⬭ 9 ⬭ 10

   Kein Schmerz — Extremer Schmerz

Beschreibung der Funktion:

Füllen Sie das Oval neben derjenigen Zahl aus, welche am besten beschreibt, wie **Schmerz** Ihren Hund innerhalb der letzten 7 Tage **beeinträchtigt hat**, hinsichtlich:

5. **Allgemeine Aktivität**

   ⬭ 0 ⬭ 1 ⬭ 2 ⬭ 3 ⬭ 4 ⬭ 5 ⬭ 6 ⬭ 7 ⬭ 8 ⬭ 9 ⬭ 10

   Nicht beeinträchtigt — Komplett beeinträchtigt

6. **Lebensgenuss**

   ⬭ 0 ⬭ 1 ⬭ 2 ⬭ 3 ⬭ 4 ⬭ 5 ⬭ 6 ⬭ 7 ⬭ 8 ⬭ 9 ⬭ 10

   Nicht beeinträchtigt — Komplett beeinträchtigt

7. **Fähigkeit aus dem Liegen in den Stand aufzustehen**

   ⬭ 0 ⬭ 1 ⬭ 2 ⬭ 3 ⬭ 4 ⬭ 5 ⬭ 6 ⬭ 7 ⬭ 8 ⬭ 9 ⬭ 10

   Nicht beeinträchtigt — Komplett beeinträchtigt

8. **Fähigkeit zu gehen**

   ⬭ 0 ⬭ 1 ⬭ 2 ⬭ 3 ⬭ 4 ⬭ 5 ⬭ 6 ⬭ 7 ⬭ 8 ⬭ 9 ⬭ 10

   Nicht beeinträchtigt — Komplett beeinträchtigt

9. **Fähigkeit zu laufen**

   ⬭ 0 ⬭ 1 ⬭ 2 ⬭ 3 ⬭ 4 ⬭ 5 ⬭ 6 ⬭ 7 ⬭ 8 ⬭ 9 ⬭ 10

   Nicht beeinträchtigt — Komplett beeinträchtigt

10. **Fähigkeit, hinaufzusteigen (zum Beispiel Treppen oder Bordsteinkanten)**

    ⬭ 0 ⬭ 1 ⬭ 2 ⬭ 3 ⬭ 4 ⬭ 5 ⬭ 6 ⬭ 7 ⬭ 8 ⬭ 9 ⬭ 10

    Nicht beeinträchtigt — Komplett beeinträchtigt

Gesamteindruck:

11. Füllen Sie das Oval neben derjenigen Antwort aus, welche die insgesamte Lebensqualität Ihres Hundes in den letzten 7 Tagen am besten beschreibt.

    ⬭ Schlecht ⬭ Ausreichend ⬭ Gut ⬭ Sehr gut ⬭ Exzellent

Canine Brief Pain Inventory (CBPI) - Hungary

A fájdalom leírása:
Osztályozza a kutya fájdalmát.

1. Azon szám melletti ovális részt jelölje be, amely a legjobban leírja a **legerősebb** fájdalmat az elmúlt 7 napban.

   ○ 0 ○ 1 ○ 2 ○ 3 ○ 4 ○ 5 ○ 6 ○ 7 ○ 8 ○ 9 ○ 10

   Nincs fájdalom — Extrém fájdalom

2. Azon szám melletti ovális részt jelölje be, amely a legjobban leírja a **legenyhébb** fájdalmat az elmúlt 7 napban.

   ○ 0 ○ 1 ○ 2 ○ 3 ○ 4 ○ 5 ○ 6 ○ 7 ○ 8 ○ 9 ○ 10

   Nincs fájdalom — Extrém fájdalom

3. Azon szám melletti ovális részt jelölje be, amely a legjobban leírja az **átlagos** fájdalmat az elmúlt 7 napban.

   ○ 0 ○ 1 ○ 2 ○ 3 ○ 4 ○ 5 ○ 6 ○ 7 ○ 8 ○ 9 ○ 10

   Nincs fájdalom — Extrém fájdalom

4. Azon szám melletti ovális részt jelölje be, amely a legjobban leírja az **éppen érzett** fájdalmat.

   ○ 0 ○ 1 ○ 2 ○ 3 ○ 4 ○ 5 ○ 6 ○ 7 ○ 8 ○ 9 ○ 10

   Nincs fájdalom — Extrém fájdalom

Funkció leírása:
Azon szám melletti ovális részt jelölje be, amely a legjobban leírja, hogy az elmúlt 7 napban a **fájdalom mennyire volt hatással** a kutyájára:

5. **Általános aktivitás**

   ○ 0 ○ 1 ○ 2 ○ 3 ○ 4 ○ 5 ○ 6 ○ 7 ○ 8 ○ 9 ○ 10

   Nem volt hatással — Nagyon komoly hatása volt

6. **Élet élvezete**

   ○ 0 ○ 1 ○ 2 ○ 3 ○ 4 ○ 5 ○ 6 ○ 7 ○ 8 ○ 9 ○ 10

   Nem volt hatással — Nagyon komoly hatása volt

7. **Fekvésből történő felállás**

   ○ 0 ○ 1 ○ 2 ○ 3 ○ 4 ○ 5 ○ 6 ○ 7 ○ 8 ○ 9 ○ 10

   Nem volt hatással — Nagyon komoly hatása volt

8. **Séta**

   ○ 0 ○ 1 ○ 2 ○ 3 ○ 4 ○ 5 ○ 6 ○ 7 ○ 8 ○ 9 ○ 10

   Nem volt hatással — Nagyon komoly hatása volt

9. **Futás**

   ○ 0 ○ 1 ○ 2 ○ 3 ○ 4 ○ 5 ○ 6 ○ 7 ○ 8 ○ 9 ○ 10

   Nem volt hatással — Nagyon komoly hatása volt

10. **Fellépés (például lépcsőre vagy járdaszegélyre)**

    ○ 0 ○ 1 ○ 2 ○ 3 ○ 4 ○ 5 ○ 6 ○ 7 ○ 8 ○ 9 ○ 10

    Nem volt hatással — Nagyon komoly hatása volt

Általános benyomás:

11. Azon megállapítás melletti ovális részt jelölje be, amely a legjobban leírja kutyája teljes életminőségét az elmúlt 7 napban.

    ○ Rossz ○ Elég jó ○ Jó ○ Nagyon jó ○ Kitűnő

Canine Brief Pain Inventory (CBPI) - Ireland

Description of Pain:
Rate your dog's pain.

1. Fill in the oval next to the one number that best describes the pain at its **worst** in the last 7 days.

   ○ 0 ○ 1 ○ 2 ○ 3 ○ 4 ○ 5 ○ 6 ○ 7 ○ 8 ○ 9 ○ 10

   No Pain — Extreme Pain

2. Fill in the oval next to the one number that best describes the pain at its **least** in the last 7 days.

   ○ 0 ○ 1 ○ 2 ○ 3 ○ 4 ○ 5 ○ 6 ○ 7 ○ 8 ○ 9 ○ 10

   No Pain — Extreme Pain

3. Fill in the oval next to the one number that best describes the pain at its **average** in the last 7 days.

   ○ 0 ○ 1 ○ 2 ○ 3 ○ 4 ○ 5 ○ 6 ○ 7 ○ 8 ○ 9 ○ 10

   No Pain — Extreme Pain

4. Fill in the oval next to the one number that best describes the pain as it is **right now**.

   ○ 0 ○ 1 ○ 2 ○ 3 ○ 4 ○ 5 ○ 6 ○ 7 ○ 8 ○ 9 ○ 10

   No Pain — Extreme Pain

Description of Function:
Fill in the oval next to the one number that best describes how during the past 7 days **pain has interfered** with your dog's:

5. **General Activity**

   ○ 0 ○ 1 ○ 2 ○ 3 ○ 4 ○ 5 ○ 6 ○ 7 ○ 8 ○ 9 ○ 10

   Does not interfere — Completely interferes

6. **Enjoyment of Life**

   ○ 0 ○ 1 ○ 2 ○ 3 ○ 4 ○ 5 ○ 6 ○ 7 ○ 8 ○ 9 ○ 10

   Does not interfere — Completely interferes

7. **Ability to Rise to Standing from Lying Down**

   ○ 0 ○ 1 ○ 2 ○ 3 ○ 4 ○ 5 ○ 6 ○ 7 ○ 8 ○ 9 ○ 10

   Does not interfere — Completely interferes

8. **Ability to Walk**

   ○ 0 ○ 1 ○ 2 ○ 3 ○ 4 ○ 5 ○ 6 ○ 7 ○ 8 ○ 9 ○ 10

   Does not interfere — Completely interferes

9. **Ability to Run**

   ○ 0 ○ 1 ○ 2 ○ 3 ○ 4 ○ 5 ○ 6 ○ 7 ○ 8 ○ 9 ○ 10

   Does not interfere — Completely interferes

10. **Ability to Climb Up (for example Stairs or Curbs)**

    ○ 0 ○ 1 ○ 2 ○ 3 ○ 4 ○ 5 ○ 6 ○ 7 ○ 8 ○ 9 ○ 10

    Does not interfere — Completely interferes

Overall Impression:

11. Fill in the oval next to the one response that best describes your dog's overall quality of life over the last 7 days.

    ○ Poor ○ Fair ○ Good ○ Very Good ○ Excellent

Canine Brief Pain Inventory (CBPI) - Japan

痛みについての説明:
あなたのイヌの痛みを評定してください。

1. 過去 7 日間で**最大**の痛みに最もよくあてはまる数字を一つ選び、隣の丸を塗りつぶしてください。
   ○0 ○1 ○2 ○3 ○4 ○5 ○6 ○7 ○8 ○9 ○10
   痛みなし　極度の痛み
2. 過去 7 日間で**最小**の痛みに最もよくあてはまる数字を一つ選び、隣の丸を塗りつぶしてください。
   ○0 ○1 ○2 ○3 ○4 ○5 ○6 ○7 ○8 ○9 ○10
   痛みなし　極度の痛み
3. 過去 7 日間の**平均的な**痛みに最もあてはまる数字を一つ選び、隣の丸を塗りつぶしてください。
   ○0 ○1 ○2 ○3 ○4 ○5 ○6 ○7 ○8 ○9 ○10
   痛みなし　極度の痛み
4. **今現在**起こっている痛みに最もよくあてはまる数字を一つ選び、隣の丸を塗りつぶしてください。
   ○0 ○1 ○2 ○3 ○4 ○5 ○6 ○7 ○8 ○9 ○10
   痛みなし　極度の痛み

機能についての説明:
過去 7 日間で**痛みが**あなたのイヌの以下をどのくらい**妨げた**かを最もよくあらわしている数字を一つ選び、隣の丸を塗りつぶしてください:

5. **全般的な活動**
   ○0 ○1 ○2 ○3 ○4 ○5 ○6 ○7 ○8 ○9 ○10
   妨げられていない　完全に妨げられている
6. **生きていることの楽しみ**
   ○0 ○1 ○2 ○3 ○4 ○5 ○6 ○7 ○8 ○9 ○10
   妨げられていない　完全に妨げられている
7. **横になっている状態から立ち上がる能力**
   ○0 ○1 ○2 ○3 ○4 ○5 ○6 ○7 ○8 ○9 ○10
   妨げられていない　完全に妨げられている
8. **歩く能力**
   ○0 ○1 ○2 ○3 ○4 ○5 ○6 ○7 ○8 ○9 ○10
   妨げられていない　完全に妨げられている
9. **走る能力**
   ○0 ○1 ○2 ○3 ○4 ○5 ○6 ○7 ○8 ○9 ○10
   妨げられていない　完全に妨げられている
10. **登りあがる能力(例えば階段や縁石)**
   ○0 ○1 ○2 ○3 ○4 ○5 ○6 ○7 ○8 ○9 ○10
   妨げられていない　完全に妨げられている

全般的な印象:

11. 過去 7 日間にわたるあなたのイヌの全般的な生活の質に最もよくあてはまる回答を一つ選び、隣の丸を塗りつぶしてください。
   ○悪い ○普通 ○良い ○非常に良い ○最高

Canine Brief Pain Inventory (CBPI) - Netherlands

Beschrijving van de pijn:
Beoordeel de pijn van uw hond.

1. Kleur het rondje in naast het cijfer dat het beste de **<u>ergste</u>** pijn in de afgelopen 7 dagen weergeeft.

   O 0 O 1 O 2 O 3 O 4 O 5 O 6 O 7 O 8 O 9 O 10

   Geen pijn — Extreme pijn

2. Kleur het rondje in naast het cijfer dat het beste de **<u>minste</u>** pijn in de afgelopen 7 dagen weergeeft.

   O 0 O 1 O 2 O 3 O 4 O 5 O 6 O 7 O 8 O 9 O 10

   Geen pijn — Extreme pijn

3. Kleur het rondje in naast het cijfer dat het beste de **<u>gemiddelde</u>** pijn in de afgelopen 7 dagen weergeeft.

   O 0 O 1 O 2 O 3 O 4 O 5 O 6 O 7 O 8 O 9 O 10

   Geen pijn — Extreme pijn

4. Kleur het rondje in naast het cijfer dat het beste de pijn **<u>op dit moment</u>** weergeeft.

   O 0 O 1 O 2 O 3 O 4 O 5 O 6 O 7 O 8 O 9 O 10

   Geen pijn — Extreme pijn

Beschrijving van het functioneren:
Kleur het rondje in naast het cijfer dat het beste weergeeft hoe de **<u>pijn</u>** in de afgelopen 7 dagen **<u>invloed had</u>** op uw honds:

5. **Algemene activiteit**

   O 0 O 1 O 2 O 3 O 4 O 5 O 6 O 7 O 8 O 9 O 10

   Geen invloed — Beïnvloedt volledig

6. **Levensvreugde**

   O 0 O 1 O 2 O 3 O 4 O 5 O 6 O 7 O 8 O 9 O 10

   Geen invloed — Beïnvloedt volledig

7. **Vermogen om vanuit liggende positie op te staan**

   O 0 O 1 O 2 O 3 O 4 O 5 O 6 O 7 O 8 O 9 O 10

   Geen invloed — Beïnvloedt volledig

8. **Vermogen om te lopen**

   O 0 O 1 O 2 O 3 O 4 O 5 O 6 O 7 O 8 O 9 O 10

   Geen invloed — Beïnvloedt volledig

9. **Vermogen om te rennen**

   O 0 O 1 O 2 O 3 O 4 O 5 O 6 O 7 O 8 O 9 O 10

   Geen invloed — Beïnvloedt volledig

10. **Vermogen om te klimmen (bijvoorbeeld trappen lopen of de stoep op)**

   O 0 O 1 O 2 O 3 O 4 O 5 O 6 O 7 O 8 O 9 O 10

   Geen invloed — Beïnvloedt volledig

Algemene indruk:

11. Kleur het rondje in naast het cijfer dat het beste weergeeft wat de algehele kwaliteit van leven is van uw hond in de afgelopen 7 dagen.

   O Slecht O Redelijk O Goed O Erg goed O Uitstekend

Canine Brief Pain Inventory (CBPI) - Portugal

Descrição da dor:
Classifique a dor do seu cão.

1. Preencha o oval junto ao número que melhor descreve o nível de dor **mais forte** nos últimos 7 dias.

O 0 O 1 O 2 O 3 O 4 O 5 O 6 O 7 O 8 O 9 O 10

Sem dor — Dor extrema

2. Preencha o oval junto ao número que melhor descreve o nível de dor **mais fraco** nos últimos 7 dias.

O 0 O 1 O 2 O 3 O 4 O 5 O 6 O 7 O 8 O 9 O 10

Sem dor — Dor extrema

3. Preencha o oval junto ao número que melhor descreve o nível de dor **médio** nos últimos 7 dias.

O 0 O 1 O 2 O 3 O 4 O 5 O 6 O 7 O 8 O 9 O 10

Sem dor — Dor extrema

4. Preencha o oval junto ao número que melhor descreve o nível de dor **atual**.

O 0 O 1 O 2 O 3 O 4 O 5 O 6 O 7 O 8 O 9 O 10

Sem dor — Dor extrema

Descrição da função:
Preencha o oval junto ao número que melhor descreve a forma como, nos últimos 7 dias, **a dor interferiu** em seu cão, relacionado a:

5. **Atividade geral**

O 0 O 1 O 2 O 3 O 4 O 5 O 6 O 7 O 8 O 9 O 10

Não interferiu — Interferiu completamente

6. **Aproveitamento da vida**

O 0 O 1 O 2 O 3 O 4 O 5 O 6 O 7 O 8 O 9 O 10

Não interferiu — Interferiu completamente

7. **Capacidade para se erguer e ficar em pé partindo da posição deitada**

O 0 O 1 O 2 O 3 O 4 O 5 O 6 O 7 O 8 O 9 O 10

Não interferiu — Interferiu completamente

8. **Capacidade para andar**

O 0 O 1 O 2 O 3 O 4 O 5 O 6 O 7 O 8 O 9 O 10

Não interferiu — Interferiu completamente

9. **Capacidade para correr**

O 0 O 1 O 2 O 3 O 4 O 5 O 6 O 7 O 8 O 9 O 10

Não interferiu — Interferiu completamente

10. **Capacidade para subir (por exemplo, escadas ou calçada)**

O 0 O 1 O 2 O 3 O 4 O 5 O 6 O 7 O 8 O 9 O 10

Não interferiu — Interferiu completamente

Impressão geral:

11. Preencha o oval junto à resposta que melhor descreve a qualidade de vida geral do seu cão nos últimos 7 dias.

O Fraca O Razoável O Boa O Muito boa O Excelente
